# IFN-I Mediates Lupus Nephritis From the Beginning to Renal Fibrosis

**DOI:** 10.3389/fimmu.2021.676082

**Published:** 2021-04-20

**Authors:** Xuewei Ding, Yi Ren, Xiaojie He

**Affiliations:** ^1^ Institute of Pediatrics, The Second Xiangya Hospital, Central South University, Changsha, China; ^2^ Laboratory of Pediatric Nephrology, Institute of Pediatrics, The Second Xiangya Hospital, Central South University, Changsha, China; ^3^ Pediatric Internal Medicine Department, Haikou Maternal and Child Health Hospital, Haikou, China

**Keywords:** fibrosis, IFN-I, lupus nephritis, nucleic acid sensors, pathogenesis, renal resident cells

## Abstract

Lupus nephritis (LN) is a common complication of systemic lupus erythematosus (SLE) and a major risk factor for morbidity and mortality. The abundant cell-free nucleic (DNA/RNA) in SLE patients, especially dsDNA, is a key substance in the pathogenesis of SLE and LN. The deposition of DNA/RNA-immune complexes (DNA/RNA-ICs) in the glomerulus causes a series of inflammatory reactions that lead to resident renal cell disturbance and eventually renal fibrosis. Cell-free DNA/RNA is the most effective inducer of type I interferons (IFN-I). Resident renal cells (rather than infiltrating immune cells) are the main source of IFN-I in the kidney. IFN-I in turn damages resident renal cells. Not only are resident renal cells victims, but also participants in this immunity war. However, the mechanism for generation of IFN-I in resident renal cells and the pathological mechanism of IFN-I promoting renal fibrosis have not been fully elucidated. This paper reviews the latest epidemiology of LN and its development process, discusses the mechanism for generation of IFN-I in resident renal cells and the role of IFN-I in the pathogenesis of LN, and may open a new perspective for the treatment of LN.

## Introduction

Systemic lupus erythematosus (SLE) is an autoimmune disease in which immune complexes (ICs) form and deposit in many organs. The kidney is one of the main target organs. Lupus nephritis (LN) is present in at least 30% to 60% of SLE patients, and almost all patients have pathologic renal involvement. The incidence of SLE and LN varies widely between regions of the world and between ethnic groups (1). Although SLE is more prevalent in women than men across all age groups and populations, several studies have shown that men with lupus more get LN than women with lupus and patients with LN are younger, mostly of African, Asian, and Hispanic race/ethnicity (2–5). LN has a mortality rate six times higher than that of the general population (6). LN is a major risk factor for SLE mortality, with 10% of patients with LN developing the end-stage renal disease (ESRD) (1, 7). Compared with SLE patients without LN, LN patients had a higher standard mortality rate (6-6.8 versus 2.4) and earlier time of death (6, 8–10). In recent years, early diagnosis, standardized treatment, and new immunosuppressants such as mycophenolate mofetil, anti-CD20 monoclonal antibody, belimumab, and other drugs have significantly improved LN prognosis. However, the 5-year mortality rate in patients with severe refractory LN remains high (1, 3, 11, 12). Therefore, elucidating its pathogenesis can provide a theoretical basis for the screening of effective therapeutic targets for LN.

IFN-I is a central factor in the occurrence and development of SLE. Recent studies suggest that IFN-I may play a role at the level of terminal organs in SLE, especially LN. IFN-I is a response to the activation of most immune cells. At present, studies on the relationship between IFN-I and LN mainly focus on immune cells in serum and kidney. Resident renal cells also have immune functions and are involved in the immune war. Previous literature has shown that resident renal cells (rather than infiltrating immune cells) are the major source of IFN-I in the kidney and that IFN-I can cause renal injury. However, there are few studies on the production of IFN-I in the kidney and the damage of IFN-I to resident renal cells. This paper reviews the relationship between IFN-I and LN resident renal cells and explores the related pathways of IFN-I promoting the pathogenesis of LN.

## Pathogenesis of LN

### IC Deposition

Nucleic acid exposure, the production of nephrogenic pathogenic antibodies and the formation of ICs are the key links leading to LN. Three mechanisms have been proposed for ICs formation or deposition on the glomerulus, and they include (1) the deposition of preformed circulating immune complexes (CICs) in the kidney, (2) the formation of in-situ ICs in the glomerulus, and (3) binding of anti-dsDNA antibodies to cross-reactive antigens present either on the surface of resident renal cells or in the extracellular environment (13–17).

Circulating autoantigens and antibodies form CICs, which are deposited in the kidney. Due to improper clearance of necrotic, apoptotic cells and/or abnormal increase in cell death in SLE patients, undegraded nucleosomes (complexes of DNA and histone-containing pairs of histone peptides) are released into the bloodstream, increasing circulating autoantigens and subsequent antibodies, which form CICs. They evade recognition by the immune system and are deposited in the kidney.

ICs can also be formed in situ. Electron-dense structures (EDS) associated with glomerular basement membrane (GBM) and the mesangial matrix constitute the main target for *in situ*-bound antibodies in both murine and human lupus nephritis. Nucleosomes and chromatin fragments accumulate due to the loss of intrarenal and extrarenal deoxyribonuclease 1 (Dnase-1) activity. Then nucleosomes and chromatin fragments readily stimulate TLR9 in infiltrating macrophages and dendritic cells, triggering the secretion of local MMPs (18, 19). MMPs degrades the membrane barrier, allowing nucleosomes and chromatin fragments to bind to GBM (20, 21). Exposure to glomerular chromatin *in situ* induces anti-chromatin (anti-dsDNA and anti-nucleosome) antibodies to become nephrogenic and pathogenic, secondary to the formation of *in-situ* ICs (15).

In addition to binding to DNA fragments, they also bind to cross-reactive antigens on the surface of renal cells to activate cell proliferation, apoptosis, inflammation and fibrosis pathways (13, 14, 17). Anti-dsDNA antibodies bind to renal mesangial cells (RMCs) through cross-reacting with the cell surface annexin II (22), α-actinin (23, 24), and ribosomal P protein (25). Anti-dsDNA antibodies bind to glomerular endothelial cells (GECs) through cross-reacting with membrane proteins with M.W. of 30–35, 44, 68, 110, and 180 kDa (26). Anti-dsDNA antibodies bind to renal tubular epithelial cells (TECs) through cross-reacting with A and D SnRNP polypeptides (27). The polyreactivity of anti-dsDNA antibodies may be related to structural/conformational similarity or molecular simulation (28). Upon binding to the cell surface, anti-dsDNA antibodies migrate to the cytoplasm and/or nucleus, promoting cell growth and proliferation, or in turn inducing apoptosis (29). Recent studies have reported that the RG2 extract from intestinal symbiotic bacterium R. gnavus cross-reacts with anti-dsDNA antibodies to trigger or exacerbate the immune pathogenesis of LN (30, 31).

**Figure 1:**
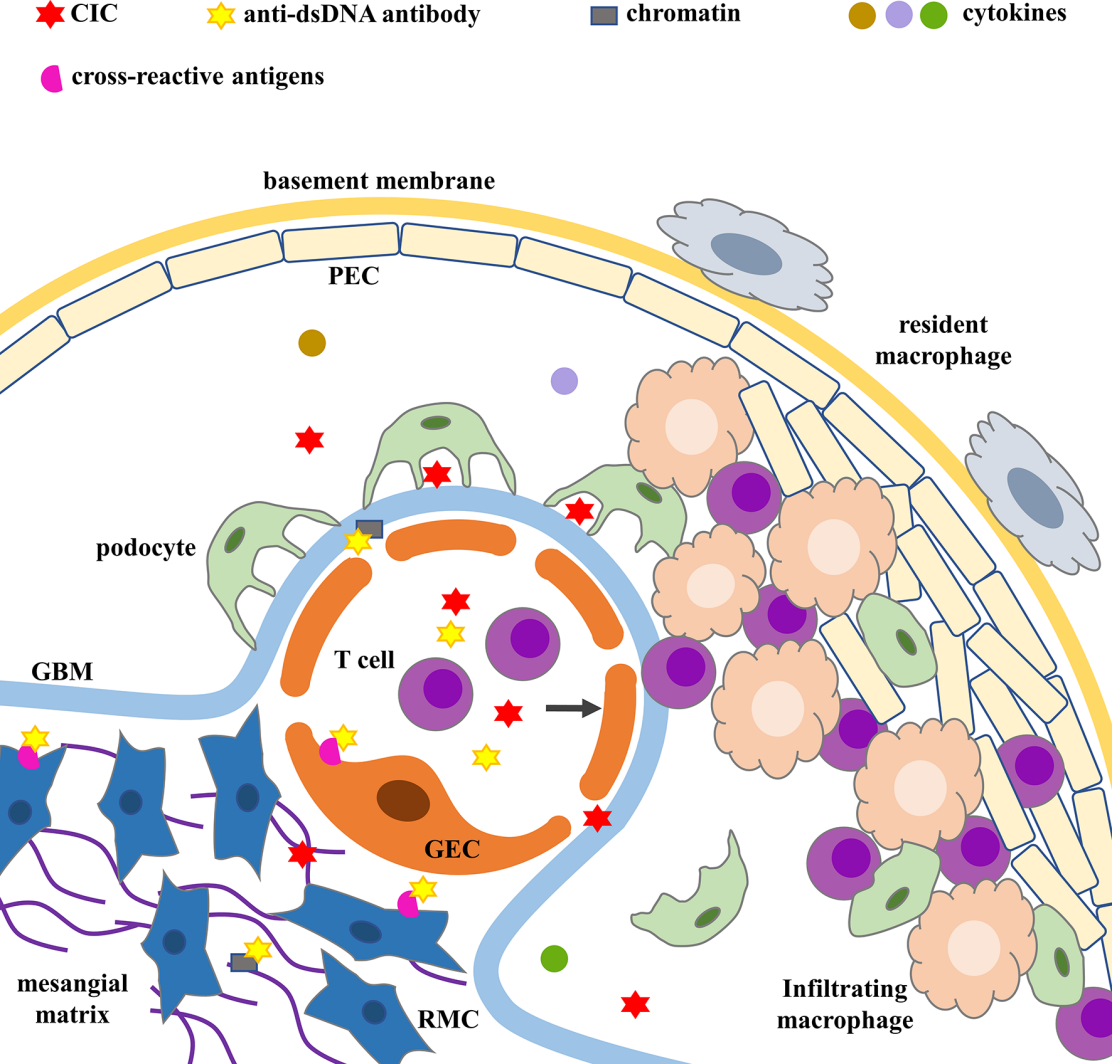
ICs deposition and glomerular injury. Cross-reactive antigens include annexin II, α-actinin, ribosomal P protein on RMCs’ surface and membrane proteins with M.W. of 30–35, 44, 68, 110, and 180 kDa on GECs’ surface. CIC, circulating immune complex; GBM, glomerular basement membrane; GEC, glomerular endothelial cell; PEC, parietal epithelial cell; RMC, renal mesangial cell.

Depending on the type, duration, and severity of LN, ICs can be found in the subendothelial, subepithelial, mesangial, and tubulointerstitial regions (**Figure 1**). The distribution, quantity, and proinflammatory properties of ICs in renal parenchyma determine complement activation, inflammation, cell proliferation, and the severity of glomerular and tubulointerstitial injuries (3, 5, 32, 33).

### Glomerulus Loss

ICs are mainly deposited in the glomerulus. The main mediator of IC-induced glomerular injury is the complement system, especially the formation of the C5b-9 membrane attack complex. C5b-9 is inserted into the glomerular membrane in extremely low amounts, transforming normal cells into inflammatory effector cells (34). Immunostimulatory glomerular cells produce large amounts of pro-inflammatory cytokines (35, 36), accelerating cell damage/aging, which may be one of the mechanisms of glomerular injury in LN (37).

The initial IC-mediated glomerular injury varies with the location of the IC deposition. IC subendothelial deposition leads to the accumulation of proinflammatory cells, causing proliferative disease and glomerular crescent (38). The GEC and the GEC surface layer (also known as the glycocalyx) are the first points of contact with the components of the circulating immune system. T cells are recruited to the glomerulus *via* the direct binding of their CD44 to the hyaluronic acid (HA) component of GEC glycocalyx (39). ICs alter cell morphology, up-regulate active caspase-3’ expression, inhibit angiogenesis, and increase NO production in GECs (40). Autophagy is a conserved metabolism that plays a protective role in many cell types and diseases. ICs inhibit the autophagy activity of GECs through Akt/mTOR-dependent pathway (41). LN antibodies promote increased secretion of endothelin-1 by GECs, leading to disruption of tight intercellular junctions (42). IC subepithelial deposition leads to podocytes damage and varying degrees of proteinuria. Podocyte injury is characterized by the foot process effacement (FPE), loss of podocyte-specific markers and cell detachment (43). Podocytes also contribute to glomerular crescent formation. Dedifferentiated podocytes migrate to cellular crescents. Podocyte injury ultimately leads to the activation and proliferation of parietal epithelial cells (PECs) through the JAK/STAT pathway, the production of HB-EGF and IL-6, and/or absence of (C-X-C motif) ligand (CXCL) 12, jointly contributing to glomerular crescent formation (43). LN IgG stimulates cellular cytoskeletal rearrangement and decreases vascular endothelial growth factor (VEGF) levels in podocytes (42). IC mesangial deposition leads to RMC proliferation and mesangial matrix increase. The inflammatory environment of LN induces RMCs to produce pro-inflammatory cytokines, which recruit leukocytes (44); promotes RMCs to express higher levels of matrix proteins and regulate matrix degradation enzymes, which lead to mesangial matrix deposition (44, 45); regulate the cell cycle and promote RMC proliferation (46).

Podocytes, GECs, and RMCs in the glomerulus interact with and support each other. Podocytes produce VEGF needed for GECs survival (47, 48); GECs produce platelet-derived growth factor (PDGF) needed for RMCs survival; RMCs isolate the potential transforming growth factor-β (TGF-β), thereby protecting GECs from apoptosis (49). Progressive injury to one cell type can eventually lead to damage of the other cell types. The activation, dedifferentiation, or proliferation of glomerular cells leads to the loss of structural integrity of the glomerular cluster and ultimately to glomerular death.

### Tubulointerstitial Fibrosis

Renal tubulointerstitium blood supply is provided by glomerular runoff. Glomerular loss affects tubulointerstitial survival. Changes resulting from loss of tubular interstitial viability, such as tubular atrophy, fibrosis, and interstitial infiltration. Injury of renal tubular epithelial cells (TECs) is an important cause of renal fibrosis (50, 51). The severity and frequency of TECs injury determine whether this repair mechanism leads to recovery or progression to fibrosis (52). TECs performs the repair mechanism to restore normal function when the injury is minor or for a short time. TECs experience maladaptive repair when severe and persistent injury exceeds the normal repair mechanism. The maladaptive repair is manifested in two aspects: cell cycle arresting in the G2/M phase, which is characterized by the expression of p53, p21 and p16^INK4a^; aging-associated secretory phenotypes, which is characterized by the secretion of pro-inflammatory factors and pro-fibrosis factors, including TGF-β1, connective tissue growth factor (CTGF), CXCL1, IL-6, IL-8 (50, 53–56). These factors promote a chronic inflammatory microenvironment conducive to fibrous tissue (53). TECs secret pro-inflammatory cytokines to recruit and activate different inflammatory cells. And these recruited cells further produce cytokines that drive the transformation of TECs, fibroblasts, and pericytes to myofibroblast type (50, 57, 58). Eventually, TECs, fibroblasts, and pericytes express α-smooth muscle actin (α-SMA) and promoting the deposition of extracellular matrix (ECM), contributing to the final process of renal fibrosis.

Although ICs are predominantly detected in the glomerulus affecting glomerular and tubulointerstitial capacity, about 70% of LN patients also have ICs aggregates along the tubular basement membrane resulting in tubulointerstitial inflammation and fibrosis. A study of LN biopsy found that tubular ICs are independent of circulatory and glomerular ICs (59). Anti-dsDNA antibodies have been shown to bind A and D SnRNP in TECs, causing them to be internalized and transported to the cytoplasmic and nuclear subcellular compartments, or they can remain at the cell surface where interaction with complement results in cell lysis (27). The binding of anti-dsDNA antibodies to TECs induces phenotypic changes in TECs that may promote the epithelial-to-mesenchymal transition (EMT) (60). Another study has shown that anti-dsDNA antibodies induce TECs secretion of soluble fibronectin and increase downstream TGF-β1 and collagen synthesis by prior activation of ERK, p38 MAPK, JNK, PKC-α and PKC-βII (61).

Pericytes are potential sources of myofibroblasts (50, 57, 58). Loss of pericytes leads to thinning of capillaries. Capillary thinning induced anoxia in TECs, which increases interstitial oxidative stress. Injured or hypoxic TECs secrete hypoxia-inducing factor-1α (HIF-1α) and subsequent VEGF to promote endothelial cells (ECs) survival and proliferation, increasing perivascular capillary density (62, 63). However, excessive production of VEGF promotes the formation of leaky and nonfunctional vessels, thus resulting in a hypoxic and highly oxidative environment (64). Besides, VEGF can be used as a pro-inflammatory factor to aggravate fibrosis response (64). Hypoxia has been shown to promote EMT as an important microenvironmental factor (65–68). Increased matrix strength also aggravates tubular hypoxia and the progression of EMT. The above factors form a vicious circle.

### Renal Microvascular Lesions

**Figure 2:**
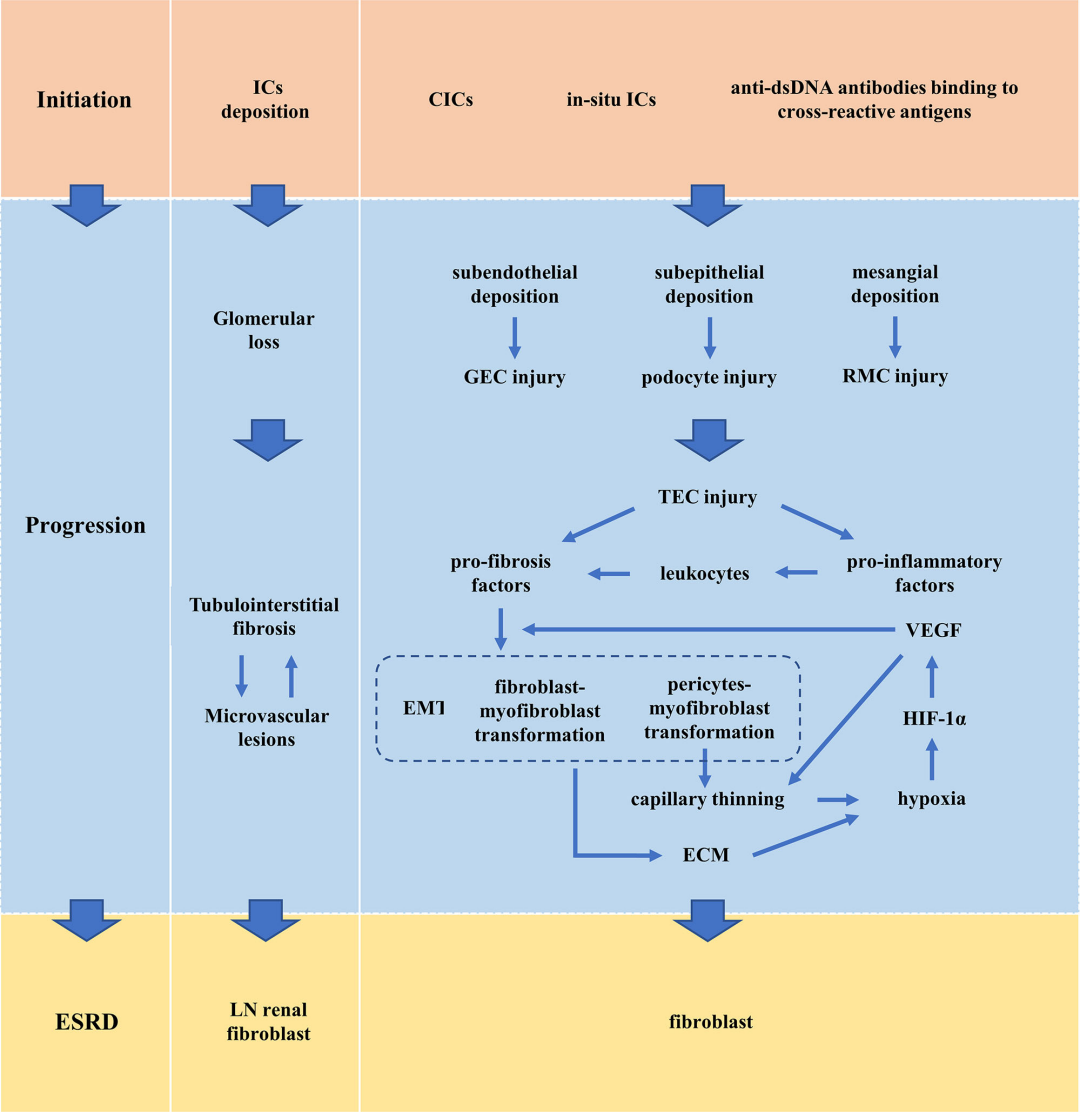
Pathogenesis of LN. CIC, circulating immune complex; ECM, extracellular matrix; EMT, epithelial-to-mesenchymal transition; ESRD, end-stage renal disease; GEC, glomerular endothelial cell; HIF-1α, hypoxia-inducing factor-1α; IC, immune complex; LN, lupus nephritis; RMC, renal mesangial cell; TEC, renal tubular epithelial cell; VEGF, vascular endothelial growth factor.

Renal microvascular lesions are common in LN and are increasingly being recognized as a marker of LN. Five pathological types of LN renal microvascular lesions have been proposed and they are vascular immune complex deposits (ICD), arteriosclerosis (AS), thrombotic microangiopathy (TMA), non-inflammatory necrotizing vasculopathy (NNV), and true renal vasculitis (TRV) (69). Up to one-third of LN patients have two or more vascular lesions at the same time. Although each lesion type may exhibit its unique factors, there are some common mechanisms among different vascular lesions. Damaged TECs induce loss of pericytes leading to thinning of capillaries (50, 57, 58, 62–64). The activation and dysfunction of vascular ECs, as well as immune system dysfunction, are key mechanisms of LN renal microvascular lesions, especially IC-induced vascular inflammation and anti-phospholipid antibody (APL)-related thrombotic events (69). The binding of autoantibodies to vascular ECs and the deposition of CICs on the microvessels lead to changes in the connections between ECs, thus activating complement, increasing the expression of adhesion molecules, inflammatory cytokines and chemokines, and increasing the permeability of ECs. The activation and dysfunction of ECs further recruit monocytes through adhesion molecules and chemokines, which induces platelet aggregation, resulting in procoagulant activity and microthrombosis (70, 71). APL-induced thrombotic events are the important mechanism of renal LN TMA (72). Patients with TMA had the worst renal outcomes (73). Renal microvascular lesions adversely affect long-term renal outcomes and may determine the selection of treatment strategies (73, 74) (**Figure 2**).

## The Mechanisms for Generation of IFN-I in the Kidney

Clinical studies have found that LN patients overexpress IFN-I, and IFN-I activity is closely related to inflammation of LN (75–77). Experimental animal studies have shown that exposure to IFN-I in NZB/W mice or C57BL/6J mice accelerates glomerulonephritis, glomerular crescent, and renal tubular interstitial nephritis (78–80); reducing the biological activity of IFN-I in NZB/W mice alleviated renal pathology and improved survival rate (81). Although a study has shown that Toll-like receptor 7 (TLR7) -mediated LN is independent of IFN-I signaling, it is not enough to mask the ultimate role of IFN-I in nephritis acceleration (82). IFN-I includes IFN-α and IFN-β, which play a biological role by binding to type I interferon receptor (IFNAR).

### The Mechanisms for Generation of IFN-I

**Figure 3:**
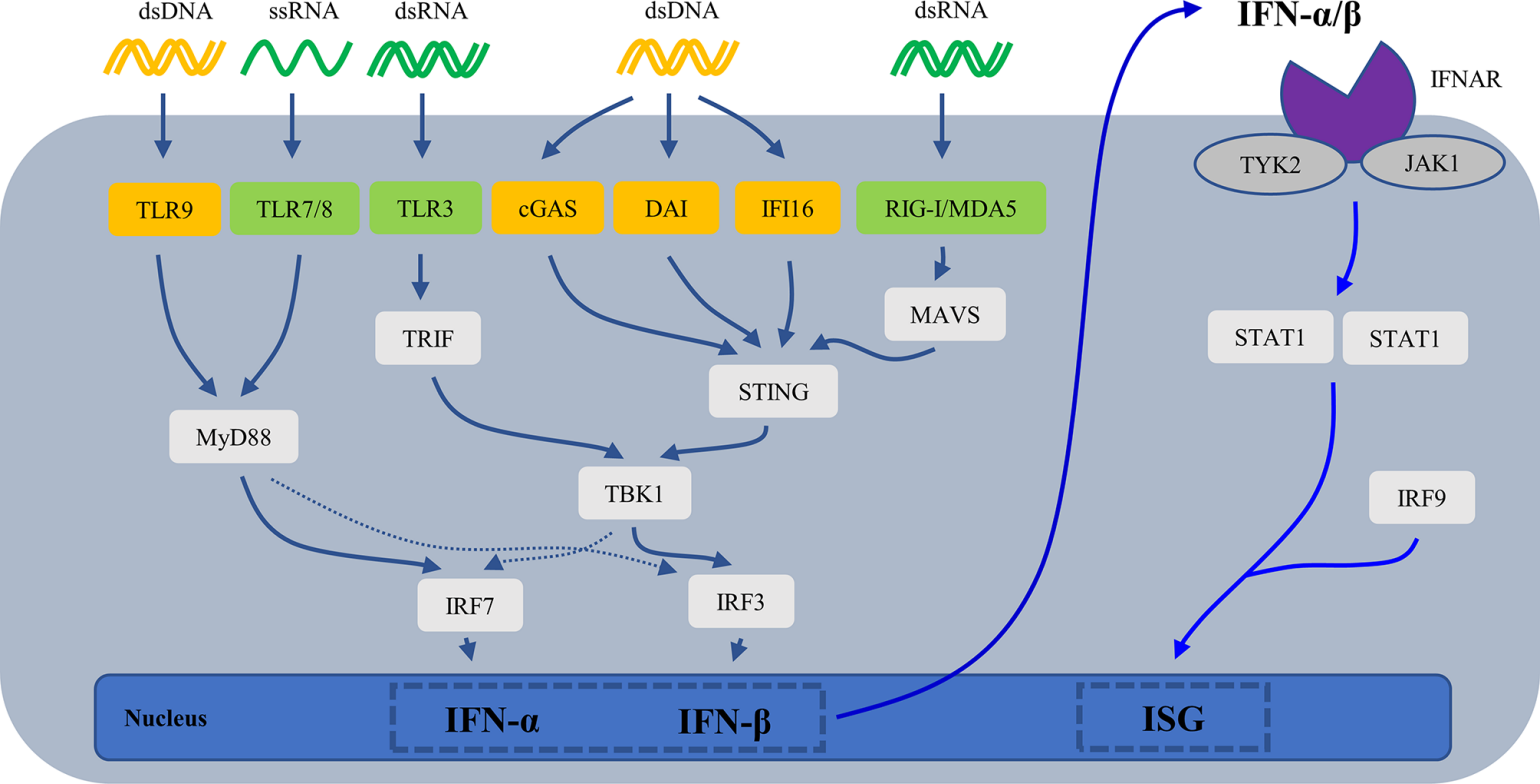
The mechanisms for generation of IFN-I. cGAS, cyclic GMP-AMP (cGAMP) synthase; DAI, DNA-dependent activator of IFN-regulatory factors; IFI16, interferon-inducible protein 16; IFN, interferons; IFNAR, type I interferon receptor; ISG, IFN-stimulated gene; MAVS, mitochondrial antiviral signaling; MDA5, melanoma differentiation-associated protein 5; RIG-I, retinoic acid-inducible gene I; STING, stimulator of interferon genes; TBK1, TANK-binding kinase 1; TLR, Toll-like receptor.

Cell-free nucleic acid (DNA/RNA) is the most effective inducer of IFN-I. They are recognized by intracellular nucleic acid sensors, which activate the signaling pathway that produces IFN-I (**Figure 3**). DNA sensors include the endosome TLR9, DNA-dependent activator of IFN-regulatory factors (DAI), interferon-inducible protein 16 (IFI16), and cyclic GMP-AMP (cGAMP) synthase (cGAS). RNA sensors include TLR3, TLR7, TLR8, retinoic acid-inducible gene I (RIG-I) and melanoma differentiation-associated protein 5 (MDA5). TLR7/8 binding with ssRNA and TLR9 binding with CpG DNA activates downstream signaling pathways—adaptor protein MyD88 and transcription factors such as IRAKs, TRAF6 and IRF7, then leading to secretion of IFN-α (83, 84).TLR3 binding with dsRNA induces IFN-β mainly through the TRIF-TBK1-IRF3 signaling pathway. cGAS (85, 86), DAI (87), IFI16 (88) recognize dsDNA and then activate the stimulator of interferon genes (STING)- TANK-binding kinase 1 (TBK1)-IRF3 signaling pathway to regulate transcription of IFN-β and IFN-induced genes. RIG-I and MDA5 recognize dsRNA and undergo conformational changes to induce mitochondrial antiviral signaling (MAVS), then activate IRF3/7 by TRAF6/3, resulting in the production of IFN-I (89).

SLE patients are rich in chromatin or cell-free nucleic acids, especially dsDNA, due to defective clearance of apoptotic cells and necrotic cells and increased neutrophil extracellular traps (NETs). These cell-free DNA/RNA acids activate above signaling pathways through intracellular DNA/RNA sensors to trigger the production of IFN-I (90). Studies have shown that there are several SLE-related susceptibility gene loci in the above signaling pathways, and their gene variants contribute to the production of IFN-I and the progression of LN (**Table 1**).

**Table 1:** Genetic variants in the DNA/RNA-IFN signaling pathway that contributes to the progression of LN.

Locus	Genetic variants	References
TLR	TLR9 (rs352140)	(91)
	TLR7 (rs385389)	(92)
	TLR5 (rs5744168)	(91)
	TLR3 (rs3775291, rs3775294)	(93)
RIG-I/MDA5	VISA (rs17857295, rs2326369)	(94)
IRF	IRF3 (rs7251)	(95)
	ITGAM (rs1143678, rs1143679, rs1143683)	(96)
NF-κB	TNIP1 (rs7708392, rs4958881)	(97, 98)
	MiR-146a (rs2431697)	(99)
STAT	STAT4 (rs7582694)	(100)

TLR9 (rs352140) (91)、TLR7 (rs385389) (92)、TLR5 (rs5744168) (91)、TLR3 (rs3775291 and rs3775294) (93)、IRF3 (rs7251) (95)、STAT4 (rs7582694) (100) are significantly associated with LN. Moreover, TNIP1 (rs7708392 and rs4958881) are associated with the risk of LN and may be involved in the disease development through abnormal regulation of NF-κB and mitogen-activated protein kinase activity (97, 98). MiR-146a (rs2431697T allele) may also increase the occurrence of LN by regulating IFN-I and NF-κB pathways (99). ITGAM variants reduce nuclear FoxO3 protein levels, thereby eliminating inhibition of IRF7 and enhancing the production of IFN-I (96, 101), which give a high risk of SLE and LN (102). Virus-induced signaling adapter (VISA) is an important adaptor protein that connects RIG-I and MDA5 with downstream signaling events. VISA rs17857295 and rs2326369 are associated with the occurrence of LN (94). MDA5, melanoma differentiation-associated protein 5; RIG-I, retinoic acid-inducible gene I; TLR, Toll-like receptor; VISA, virus-induced signaling adapter.

### The Main Producers of IFN-I in the Kidney

The IFN-I system in SLE is in a long-term activation state. All nucleated cell types can produce IFN-I during pathogenic infection. Under the background of SLE, immune cells are abnormally activated. For example, plasmacytoid dendritic cells (pDCs) massively produce IFN-α (103); neutrophils secrete IFN-I in the early stages of the disease (104). Early T1 B cells in SLE produce IFN-I, especially IFN-β (105). A previous study has shown that renal resident cells (rather than infiltrating immune cells) were the main source of IFN-I in the kidney (80). Besides the circulating cell-free nucleic acid and the nucleic acid component of CICs, renal immunostimulatory nucleic acids are an important source of pathogenic nucleic acid. Large chromatin fragments in the kidney are exposed due to selective down-regulation of Dnase1 activity in the kidney (16, 106, 107). Lupus nephrogenic autoantibodies enter renal cells, damaging cell structure, enhancing DNA cleavage, and inducing cell death (29, 108). Another potential source of renal immunostimulatory nucleic acids is NETs released by neutrophils in the glomerulus and renal tubule, which are not fully degraded and are made of DNA, histones, and neutrophil proteins (109–111). NETs activate the cGAS-STING pathway or the TLR9 pathway to produce IFN-I (111, 112). The IFN-I subtype secreted by renal resident cells and DNA/RNA receptors’ expression in renal resident cells varied (**Table 2**).

**Table 2:** Distribution and expression of DNA/RNA sensors in renal resident cells.

Renal resident cells		RMCs	GECs	Podocytes	TECs	fibroblasts	PTC ECs
DNA sensors	TLR9	N	N	Y	U	U	Y
	DAI	Y	Y	U	U	U	U
	IFI16	U	U	Y	U	U	U
	cGAS	U	U	Y	U	U	U
RNA sensors	TLR3	Y	Y	Y	U	U	U
	TLR7	N	N	N	U	U	U
	TLR8	N	N	Y	U	U	U
	RIG-I/MDA5	Y	Y	Y	Y	U	U

Y means “yes”; N means “no”; U means “unknown”. GEC, glomerular endothelial cell; PTC EC, peritubular capillary endothelial cell; RMC, renal mesangial cell; TEC, renal tubular epithelial cell.

#### Podocyte

DNA/RNA-ICs induce IFN-β production in podocytes. Podocytes treated with TLR3 ligand—polyIC—expressed IFN-I. And podocytes express TLR1-6 and TLR9 (113). Masum MA et al. found that TLR9 is overexpressed in podocytes in mice with autoimmune glomerulonephritis (AGN), which is associated with glomerular podocyte injury (114). However, Machida H et al. found that TLR9 was expressed only in podocytes from active LN patients and disappeared during remission (115). cGAS and IFI16 are the main DNA sensors in podocytes and trigger the expression of IFN-β by activating the cGAS/IFI16-STING pathway, thereby promoting the progression of LN in SLE patients (116). Besides, Kimura J et al. analyzed BXSB/MPJ-YAa lupus model mice and found that the expression of TLR8 and its downstream cytokines was significantly increased in lupus mice, and TLR8 was localized in podocytes (117).

#### RMC

DNA/RNA-ICs induce IFN-β production in RMCs. In the context of SLE, RMCs express TLR1-4 and TLR6, especially highly express TLR3 (118).TLR3 belongs to the nucleic acid-specific TLR subgroup that activates the IFN-β production by recognizing dsRNA (119). However, RMCs do not express other members of the TLR subgroup—TLR7-9 (118, 119). Besides, LN patients’ RMCs show high levels of MDA5 expression (120). dsRNA induces RMCs to release IFN-α/β by MDA5 (rather than RIG-I); IFN-α/β can activate RMCs in an autocrine-paracrine loop (121). Although RMCs do not express TLR9 (118, 119), DNA-ICs also induce RMCs activation. Qing X et al. found that IgG anti-dsDNA antibodies up-regulate RMCs pro-inflammatory genes in MRL/LPR mice (122). Allam R et al. found that viral dsDNA stimulated RMCs to produce IFN-β and IFN-induced genes which are independent of DAI (123).

#### GEC

DNA/RNA-ICs induce IFN-β production in GECs. GECs express TLR1-6 (124). dsRNA activates TLR3 and induces GECs to express IFN-β (125, 126). Liu Q et al. found that dsRNA induced GECs expression of RIG-I and MDA5 through the TLR3/IFN-β signaling pathway (127). At the same time, dsRNA activates GECs through RIG-I to secrete IFN-α/β, while IFN-α/β cannot activate GECs in an autocrine-paracrine loop (128). GECs lacks a unique DNA-specific TLR—TLR9 (124). However, Hagele H et al. stimulated GECs with viral dsDNA and found that viral dsDNA entered GECs through endocytosis and then activated GECs to produce IFN-α/β in a TLR-independent manner (129). IFN-β can induce DAI expression and IRF3 phosphorylation, but IFN-β cannot activate GECs in an autocrine-paracrine loop (129).

#### Others

Resident renal cells also include TECs, renal interstitial fibroblasts, and peritubular capillary endothelial cells (PTC ECs). Castellano G et al. found that TECs were the main producer of IFN-α (130). Recent studies have found that TECs express RIG-I, an intracellular pattern-recognition receptor that participates in the production of IFN-β by recognizing RNA (131). It is unknown whether renal interstitial fibroblasts produce IFN-I and their intracellular DNA/RNA receptor expression. The TLR9 expression level was significantly increased in PTC ECs in lupus-prone AGN model mice and was associated with peritubular capillary and renal tubular interstitial injury (132).

## The Damage Effects of IFN-I in LN

**Figure 4:**
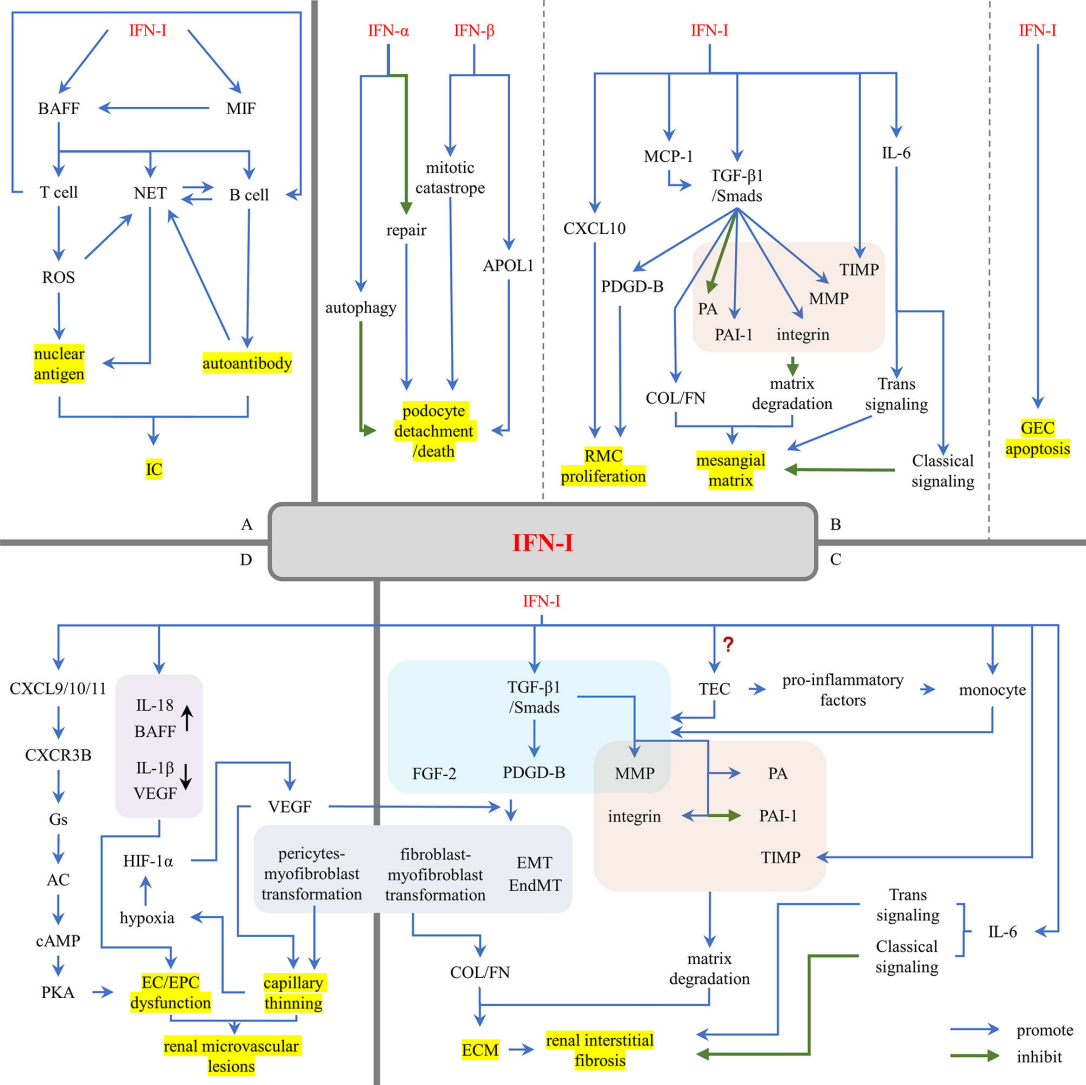
The damage effects of IFN-I in LN. **(A)** IFN-I promotes the formation of nuclear antigens and autoantibodies. **(B)** IFN-I promotes glomerular sclerosis. **(C)** IFN-I promotes renal interstitial fibrosis. **(D)** IFN-I promotes renal microvascular lesions. AC, adenylyl cyclase; APOL1, apolipoprotein L1; BAFF, B cell activating factor; cAMP, cyclic adenosine monophosphate; COL, collagen; CXCL, (C-X-C motif) ligand; CXCR3B, chemokine-receptor-3B; EC, endothelial cell; ECM, extracellular matrix; EMT, epithelial-to-mesenchymal transition; EndMT, endothelial-to-mesenchymal transition; EPC, endothelial progenitor cell; FGF-2, fibroblast growth factor 2; FN, fibronectin; GEC, glomerular endothelial cell; HIF-1α, hypoxia-inducing factor-1α; IC, immune complex; IFN-I, type I interferons; MCP-1, monocyte chemotaxis proteins 1; MIF, macrophage inhibitory factor; MMP, matrix metalloproteinases; NET, neutrophil extracellular trap; PA, plasminogen activator; PAI-1, plasminogen activator inhibitor 1; PDGD, PDGF, platelet-derived growth factor; PKA, protein kinase A; RMC, renal mesangial cell; ROS, reactive oxygen species; TEC, renal tubular epithelial cell; TIMP, tissue inhibitors of metalloproteinases; TGF-β1, transforming growth factor-β1.

Renal resident cells are the main source of IFN-I in the kidney (80). Renal resident cell-induced IFN-I, in turn, promotes the inflammatory state of glomerular cells, leading to renal fibrosis, scarring and renal loss (80). The damage of IFN-I is manifested in three aspects: (1) IFN-I induces the production of nuclear antigen and autoantibodies, promoting the formation of ICs; (2) IFN-I recruits leukocytes to promote proliferative lesions; (3) IFN-I acts on resident renal cells, leading to cell activation, injury, apoptosis, and progression to renal fibrosis (**Figure 4**).

### IFN-I Promotes the Formation of Nuclear Antigens and Autoantibodies

IFN-I promotes the formation of nuclear antigens. IFN-I can induce B cell activating factor (BAFF) expression and mobilization (133, 134). BAFF promotes the activation of T cells (135) and the production of NETs (136). Overactivity of SLE T cells leads to mitochondrial hyperpolarization, which ultimately leads to increased production of reactive oxygen species (ROS) (137). ROS can modify cellular components and metabolites, giving them immunogenicity (138). ROS contributes to the formation of NETs (112). NETs trigger a concerted activation of TLR9 and B-cell receptor (BCR) leading to autoantibodies production in lupus (139). Follicular helper T cells (TFHs) (140, 141), CXCR5-CXCR3^+^PD1hiCD4^+^T helper cells (142), and peripheral helper T cells (TPHs) (143) promote B cells differentiation and antibody production in different ways.

IFN-I promotes the formation of autoantibodies. BAFF is a key factor in the maturation, survival and function of SLE pathogenic B cells (144, 145), which are responsible for the production of autoantibodies. IFN-I not only directly mobilized BAFF (133, 134) but also indirectly regulated the BAFF pathway by promoting the production of macrophage inhibitory factor (MIF) (146–148). BAFF also promotes the activation of B cells by IFN (149). Besides, SLE-related autoantibodies and ICs can induce the strong release of NETs (150), increasing nucleic acid exposure.

Then increased nuclear antigens and autoantibodies induced by IFN-I enhance the chance of IC formation and triggers LN.

### IFN-I Promotes Leukocyte Infiltration

IFN-I strongly induces chemokine CXCL9/10/11, then recruiting leukocytes into the inflammatory site through the CXCR3A-Gi-PI3K-MAPK signaling pathway (151, 152). Several studies have found that renal IFN-I induced leukocytes into the kidney in LN patients. Increased leukocyte recruitment might be an operative mechanism that IFN-I drives immune-mediated nephritis (80). Triantafyllopoulou A et al. induced IFN-β overexpression in NZB/W mice using TLR3 ligand poly (I: C) and found that IFN-β induced macrophagic infiltration in renal tissue (78). Yoshikawa M et al. found that IFN-β down-regulates CXCR5 expression in B cells and IFN-γ upregulates CXCR3 expression in B cells, which induces B cell infiltration in renal tissue of LN patients (153). Besides, IFN-I regulates these immune cells. Kishimoto D et al. found that IFN-I inhibited the anti-inflammatory properties of M2-like macrophages in the glomerulus by up-regulating Bach1 and down-regulating ho-1 expression, thus promoting glomerular inflammation (154).

### IFN-I Promotes Renal Tissue Injury

#### IFN-I Promotes Glomerular Sclerosis

##### Podocyte

Impair to podocyte structure is one of the early symptoms of glomerular injury and is a characteristic of LN (155–157). Podocytes are highly differentiated epithelial cells that are fixed on the basement membrane through the extension of the foot process and interact with the surrounding podocytes to form a slit diaphragm and eventually a filtration barrier. The slit diaphragm is a unique cellular connection formed by podocin-specific proteins such as nephrin and podocin, which interact with the actin cytoskeleton (158). The actin cytoskeleton is the main structure of podocytes. Disorders of actin cytoskeleton play a major role in FPE and mitotic catastrophe, leading to podocytes detachment and proteinuria (159–161).

Podocyte cells are induced to produce IFN-β, which in turn stimulates podocyte B7-1 expression and actin remodeling (162). IFN-β specifically promotes podocyte detachment or death by inducing mitotic catastrophe in podocytes. IFN-α prevents podocyte repair by causing cell-cycle arrest and inhibiting proliferation and migration of PECs. And both of the above IFNs suppress renal progenitor differentiation into mature podocytes, which conducive to focal scar formation but not to glomerular repair (163). dsDNA induces podocytes to secret IFN-β. IFN-β expression activates IFNAR. IFNAR-associated JAK1 and TYK2 kinases then phosphorylate STAT1, which promotes transcription of apolipoprotein L1 (APOL1). And activated STAT1 up-regulates IFI16, which triggers a positive feedback mechanism promoting APOL1’s expression (116). Overexpression of APOL1 in podocytes is highly toxic. The APOL1 allele G1 and G2 are risk factors for LN and end-stage renal disease associated with lupus nephritis (LN-ESRD) in African Americans (164, 165). The observed injury of glomerular podocytes in LN suggests that the increase of APOL1 risk variant in podocytes of SLE patients may promote the faster progression of LN and LN-ESRD (155–157). Recent studies have shown that IFN-α is also associated with damage to podocyte structure and function. IFN-α has a significant effect on the filtration barrier function of podocytes. At the same time, IFN-α attenuates the mTORC1 signal and induces podocyte autophagy. However, increased autophagy ameliorates IFN-α-induced podocyte injury (166). This seems to show a protective negative feedback regulation.

##### GEC

GECs are also the component of the glomerular filtration barrier. Previous studies have shown that IFN-I, especially IFN-α, mediated endothelial dysfunction and caused the EC apoptosis (167), which increases GECs permeability and results in a loss of glomerular filtration barrier function.

##### RMC

RMCs are the key factor in LN glomerular fibrosis in LN. They play an important role in homeostasis by maintaining glomerular structure, producing and maintaining mesangial matrix, regulating filtration surface area and phagocytizing apoptotic cells or ICs (49). In response to ICs deposition and cytokine-induced injury, RMCs promote glomerular fibrosis through hypertrophy and proliferation (168). PDGF-B is a proliferation/migration-inducing growth factor that induces RMC proliferation in glomerulonephritis (169). TGF-β1 activates the downstream Smads signaling pathway by autocrine/paracrine, inducing the production of PDGF-B. The IFN-β autocrine/paracrine loop activates Smad7 which inhibits Smad3/4 activation and prevents induction of PDGF-B (170). However, studies have shown that IFN-α/β stimulation increases TGF-β1 expression (44, 78), which may enhance the expression of PDGF-B and promote the proliferation of RMCs. Moreover, CXCL10 induced by IFN-I not only recruit leukocytes but also aggravates RMC proliferation by activating ERK signaling pathway (171).

In addition to overproliferation, RMCs are one of the major stromal generating cells, secreting mesangial matrix components, such as type I collagen (COL I), type III collagen (COL III), and fibronectin (FN). TGF-β1/Smads signaling pathway plays a major role in the excess extracellular matrix (ECM) (172, 173). First, the TGF-β1/Smad signaling pathway up-regulated matrix protein synthesis, including COL I and COL III. Second, the TGF-β1/Smad signaling pathway inhibits matrix degradation. The addition of TGF-β1 to normal glomerulus significantly reduced the activity of plasminogen activator (PA) and increased the synthesis of plasminogen activator inhibitor 1 (PAI-1) (174). TGF-β1 regulates the expression of MMP-9 (44); the main function of MMPs is to degrade ECM components, so it seems that TGF-β1 enhances matrix degradation. However, a large number of studies have shown that the levels of MMPs and tissue inhibitors of metalloproteinases (TIMPs) in serum, urine and glomerulus of LN patients are increased, accompanied by the deposition of mesangial matrix (78, 175–179). Overexpressed MMPs interact with TIMPs, changing matrix composition to promote mesangial matrix expansion (178). IFN-α/β induces high expression of MMP-9 and TIMP-1 in the kidney (78). Besides, TGF-β1 alters the expression of mesangial α1β1 and α5β1 integrins and their ligands (such as laminin, collagen, and FN), promoting matrix adhesion (180).

IFN-I can indirectly induce TGF-β1 expression in RMCs. In addition to CXCL10, IFN-I induced RMCs expression of monocyte chemotaxis proteins 1(MCP-1/CCL2) and IL6. Increased MCP-1 levels stimulate the formation of TGF-β1 in renal resident cells (181) and induce Col IV mRNA expression, collagen deposition, and FN expression (182). The role of IL-6 in renal fibrosis remains controversial. Previous studies have shown that IL-6 does not play an important role in the development of renal fibrosis (183). Recent studies have shown that overexpression of IL-6 and its receptor reduces the abundance of FN and Col IV in RMCs (184); IL-6 trans signal transduction may be involved in the occurrence and development of renal fibrosis (185). This is consistent with the theory that IL-6 signaling is mediated through two main pathways. The anti-inflammatory activity of IL-6 is mediated through classical signaling pathways, whereas the pro-inflammatory property is mediated through trans-signaling pathways (186). Moreover, IFN-I autocrine/paracrine loops largely induce RMCs death (121). On the whole, IFN-I shows a significant damaging effect on RMCs (187, 188).

#### IFN-I Promotes Renal Interstitial Fibrosis

Renal interstitial fibrosis is the result of the chronic inflammatory process. During chronic inflammation, different cell components and complex signaling networks interact to lead to the development of renal myofibroblasts, which lead to excessive accumulation of ECM, a major and common feature of different chronic kidney diseases. The possible origin of myofibroblasts from renal epithelial/endothelial cells, fibroblasts, or pericytes remains a subject of debate (189–195). LeBleu VS et al. showed that proliferative myofibroblasts account for 50%, derived from resident fibroblasts; non-proliferative myofibroblasts derive through differentiation from bone marrow (35%), the endothelial-to-mesenchymal transition (EndMT) program (10%) and the epithelial-to-mesenchymal transition (EMT) program (5%) (193). TGF-β1 still plays a central role in many fibrotic factors (196). First, TGF-β1 promotes the proliferation of fibroblasts. fibroblast growth factor 2 (FGF-2) is a powerful mitogen of fibroblasts, promoting the autocrine growth of fibroblasts (197). TGF-β1, PDGF-B and FGF-2 jointly promote the proliferation of fibroblasts (197–199). Second, TGF-β1 promotes the transformation of other cells into myofibroblasts. TGF-β1 induces the functional transformation of TECs and GECs into myofibroblasts, which are responsible for ECM deposition (193, 200–204). MMP-9 is involved in EndMT and EMT through Notch signaling up-regulation, and its activation is located downstream of TGF-β1 (205, 206). FGF-2 also plays an important role in EMT (207–209). TGF-β1 is also involved in fibroblast-myofibroblast transformation through TGFR1 phosphorylation and subsequent Smad2/3 pathway mediating α-SMA transcription and myofibroblast differentiation (210). TGF-β1 and PDGF transform fibroblasts into myofibroblasts (211, 212), which together with fibroblasts produce ECM (213, 214). In addition to the TGF-β1 signaling pathway, the PDGF signaling induces pericytes proliferation and differentiation into myofibroblasts (64, 215–218). Moreover, TGF-β1 regulates PA, PAI-1, MMP-9, and integrin to inhibit matrix degradation and promote ECM accumulation and interstitial fibrosis (44, 174, 178, 180).

TGF-β1 is mainly produced by TECs. Whether IFN-I induces TECs to secrete TGF-β1 remains to be seen. IFN-α induces barrier instability and apoptosis of TECs (219–221), which may activate TECs. In the recent single-cell RNA sequencing studies of renal biopsies from LN patients, the expression of IFN-I response genes in TECs from LN patients was significantly higher than those of healthy control subjects (222) and correlates with clinical scores and with the response to treatment (223). Activated TECs secrete a series of pro-inflammatory mediators and absorb more circulating monocytes into the renal tubulointerstitium; infiltrated monocytes become activated macrophages (224). IFN-I also recruited macrophages to infiltrate (78). Activated macrophages secrete PDGF, TGF-β1, MMP and TIMP, which are involved in the regulation of tissue fibrosis (224). Similarly, IFN-I can enhance the process of renal interstitial fibrosis through MCP-1/CCl2 and IL-6 (181, 184, 185).

#### IFN-I Promotes Renal Microvascular Lesions

The imbalance between vascular endothelial injury and repair is a key event in vascular lesions. IFN-I breaks this balance (225). Endothelial progenitor cells (EPCs) are the main repair mechanism. IFN-I induces CXCL9/10/11 expression. CXCL9/10/11 activates chemokine-receptor-3B (CXCR3B)-Gs-adenylyl cyclase (AC)-cyclic adenosine monophosphate (cAMP)-protein kinase A (PKA) signaling pathways, directly promoting ECs and EPCs dysfunction (225). It also up-regulates the function of other pro-EPC dysfunction factors (IL-18 (226), BAFF (133, 134, 227)) and down-regulates the function of pro-angiogenic molecules (IL-1β and VEGF (167)), which indirectly leads to EPC dysfunction.

IFN-I promotes vascular imperfection by affecting pericytes. IFN-I regulates TGF-β1 and PDGF expression (44, 78, 170). TGF-β1 and PDGF signaling pathways induce pericytes to proliferate and differentiate into myofibroblasts (64, 215–218). Pericytes are attached to the surface of capillary wall and share a developmental origin with fibroblasts. Normal pericytes stabilize the walls of blood vessels and maintain vessel tranquility and integrity. Activated pericytes are shed from the vascular wall and transformed into myofibroblasts (195, 228–232). Loss of pericytes leads to the formation of fragile capillaries and unstable, pathological blood vessels, ultimately resulting in renal vascular thinning (233). The loss of capillaries around renal tubules is closely related to renal fibrosis.

## Conclusion

Cell-free DNA/RNA accumulation is the initial step of lupus and LN. Cell-free DNA/RNA and the nucleic acid components of ICs trigger the DNA/RNA sensors in renal resident cells, thus activating the signaling pathway for IFN-I production. IFN-I in turn induces nucleic acid exposure and the formation of autoantibodies. IFN-I acts on renal resident cells and is involved in the whole process of renal injury, especially the activation of the TGF-β1/Smads signaling pathway. Also, IFN-I recruits leukocytes into renal tissues through the CXCL9/10/11-CXCR3A-Gi-PI3K-MAPK signaling pathway, enhancing renal fibrosis response. Moreover, IFN-I promotes renal microvascular lesions, further damaging renal function. IFN-I is found in almost every link of the pathogenesis of LN. Therefore, IFN-I plays an important role in the pathogenesis of LN. Targeting IFN-I systems in the kidney has potential therapeutic effects on the premature emergence of LN in SLE patients. It also suggests that the immune function of renal resident cells is greater than that of the renal immune cells in LN and renal resident cells are the dominant player and acceptor in the occurrence and development of LN. The study on renal resident cells will further deepen the understanding of LN and contribute to the targeted therapy of LN in the future.

## Author Contributions

XD and YR did the literature search and drafted the article. XH gave insight. XH revised the article. All authors contributed to the article and approved the submitted version.

## Funding

This work was supported by National Natural Science Foundation of China (No. 61562021 and No. 81560275, No. 81960885, No. 81260139, No. 81060073, No. 30560161), Hainan Major Science and Technology Projects (ZDKJ2039010), Hainan Association for academic excellence Youth Science and Technology Innovation Program (201515), Hainan special projects of Social Development (ZDYF2018103 and 2015SF39).

## Conflict of Interest

The authors declare that the research was conducted in the absence of any commercial or financial relationships that could be construed as a potential conflict of interest.

## Glossary

**Table:**

AC	adenylyl cyclase
AGN	autoimmune glomerulonephritis
APOL1	apolipoprotein L1
APL	anti-phospholipid antibody
AS	arteriosclerosis
BAFF	B cell activating factor
BCR	B-cell receptor
cAMP	cyclic adenosine monophosphate
cGAS	cyclic GMP-AMP (cGAMP) synthase
CIC	circulating immune complex
COL I	type I collagen
COL III	type III collagen
CTGF	connective tissue growth factor
CXCL	(C-X-C motif) ligand
CXCR3B	chemokine-receptor-3B
DAI	DNA-dependent activator of IFN-regulatory factors
Dnase-1	deoxyribonuclease 1
EC	endothelial cell
ECM	extracellular matrix
EDS	electron-dense structures
ESRD	end-stage renal disease;
EMT	epithelial-to-mesenchymal, transition;
EndMT	endothelial-to- mesenchymal transition
EPC	endothelial progenitor cell
FGF-2	fibroblast growth factor 2
FN	fibronectin
FPE	foot process effacement
GBM	glomerular basement membrane
GEC	glomerular endothelial cell
HA	hyaluronic acid
HIF-1α	hypoxia- inducing factor-1α
IC	immune complex
ICD	vascular immune complex deposits
IFI16	interferon-inducible protein 16
IFNAR	type I interferon receptor
IFN-I	type I interferons
ISG	IFN-stimulated gene
LN	lupus nephritis
LN-ESRD	end-stage renal disease associated with lupus nephritis
MAVS	mitochondrial antiviral signaling
MCP-1	monocyte chemotaxis proteins 1
MDA5	melanoma differentiation-associated protein 5
MIF	macrophage inhibitory factor
MMP	matrix metalloproteinases
NET	neutrophil extracellular trap
NNV	non-inflammatory necrotizing vasculopathy
PA	plasminogen activator
PAI-1	plasminogen activator inhibitor 1
pDC	plasmacytoid dendritic cell
PDGF	platelet-derived growth factor
PEC	parietal epithelial cell
PKA	protein kinase A
PTC EC	peritubular capillary endothelial cell
RIG-I	retinoic acid-inducible gene I
RMC	renal mesangial cell
ROS	reactive oxygen species
SLE	systemic lupus erythematosus
STING	stimulator of interferon genes
TBK1	TANK- binding kinase 1
TEC	renal tubular epithelial cell
TFH	Follicular helper T cell
TGF- β1	transforming growth factor-β1
TIMP	tissue inhibitors of metalloproteinases
TLR	Toll-like receptor
TMA	thrombotic microangiopathy
TPH	peripheral helper T cell
TRV	true renal vasculitis
VEGF	vascular endothelial growth factor
VISA	virus- induced signaling adapter α-smooth muscle actin (α-SMA)
